# Retraction: Multidisciplinary Approaches to Limb Salvage in Traumatic Extremity Injuries From Emergency Triage to Definitive Vascular, Orthopedic, and Reconstructive Surgery: A Systematic Review

**DOI:** 10.7759/cureus.r197

**Published:** 2025-10-17

**Authors:** Nabila Issa Youssouf, Huba Ali Khan, Asma Ahmed Osman Mohamed, Mohamed Bahaa Aboelsebaa Othman, Mahmoud Hassan Fadul Ismail, Ahmed A Hassan, Aliaa H Alkhazendar, Jarallah H. J. Alkhazendar, Monezahe Zehra, Deewan Aakash

**Affiliations:** 1 Surgery, International University of Africa, Khartoum, SDN; 2 Emergency Medicine, Ziauddin University, Karachi, PAK; 3 Surgery, Burjeel Medical City, Abu Dhabi, SAU; 4 Vascular Surgery, Nasser Institute Hospital for Research and Treatment, Cairo, EGY; 5 International Public Health, Royal Infirmary of Edinburgh, Edinburgh, GBR; 6 Orthopedics and Trauma, Suez Canal University Hospital, Ismailia, EGY; 7 Surgery, Islamic University of Gaza, Gaza, PSE; 8 General and Emergency Surgery, East and North Hertfordshire NHS Trust, Lister Hospital, Stevenage, GBR; 9 Surgery, Abbasi Shaheed Hospital, Karachi, PAK; 10 Surgery, Liaquat National Hospital, Karachi, PAK

The Editors-in-Chief have retracted this article. An investigation by the journal found evidence of authorship manipulation. The Editors-in-Chief therefore no longer have confidence in the provenance and reliability of the article contents. The authors disagree with the decision to retract.

